# Supplementary material for: A Multi‐Stage Drop‐the‐Loser Design With Superiority Boundaries
Source: Stat Med. 2026 Jun 24;45(15-17):e70642. doi: 10.1002/sim.70642 (PMC13291772; doi:10.1002/sim.70642)
Supplement: Supplementary file 1 — Data S1. Supporting Information. [file SIM-45-0-s001.pdf]

Supporting Information: A Multi-Stage Drop-the-Loser Design with Superiority Boundaries

Peter Greenstreet, Manel Khan, Salmaan Kanji, Pouya Motazedian, Andrew Seely, Stephanie Sibley, Tim Ramsay

1 Equations for the proposed approach to calculate the PWER for the motivating example

For the motivating example the PWER equals

$$1 - \int_{-\infty}^{u_1} \int_{-\infty}^{u_2} \int_{-\infty}^{u_3} \phi\Big(\mathbf{z}, \mu', \Sigma'\Big) \mathbf{dz},$$

where $\phi(\mathbf{z}, \mu, \Sigma)$ is the probability density function of a multi-variate normal distribution with mean μ and covariance matrix Σ, with

$$\mu' = (0, 0, 0)$$

and

$$\Sigma' = \begin{pmatrix} 1 & \sqrt{\frac{1}{2}} & \sqrt{\frac{1}{3}} \\ \sqrt{\frac{1}{2}} & 1 & \sqrt{\frac{2}{3}} \\ \sqrt{\frac{1}{3}} & \sqrt{\frac{2}{3}} & 1 \end{pmatrix}.$$

2 Proof of Theorem 1

Proof. Let $D_{k^\star,j}$ define the event that treatment $k^\star$ is not found to be better than the control at the given stage j,

$$D_{k^\star,j} = \begin{cases} Z_{k^\star,j} \leq u_{k,j} & \text{if } k^\star \text{ is still in the trial} \\ \Omega & \text{if } k^\star \text{ is not in the trial} \end{cases}.$$

where Ω is the whole sample space. Let $m_1, \dots m_K$ be the ordering in which the treatments are dropped from the trial. With m_K being the last treatment dropped. We define $m_1 \in \{1, \dots, K\}$ and define $m_k \in \{1, \dots, K\}/\{m_1, \dots, m_{k-1}\}$. For any given $m_1, \dots m_K$ the event that treatment $k^\star$ is not dropped from the trial equals:

$$R = \bigcap_{j=1}^{J-1} \Bigg(D_{k^\star,j} \cup \bigcup_{k=\{j+1,\dots,J\}} B_{m_k,j}\Bigg) \cap \Bigg(D_{k^\star,J} \cup B_{m_K,J}\Bigg). \tag{2.1}$$

R in Equation 2.1 can be broken down into 2 key parts. The first part is $D_{k^\star,j} \cup \bigcup_{k=\{j+1,\ldots,J\}} B_{m_k,j}$. This states that at each stage (up-to stage J-1) the trial does not stop for superiority. For stage J this is given by the equation $D_{k^\star,J} \cup B_{m_K,J}$. The second part is the intercept of all these events is calculated. This is because for treatment $k^\star$ not to be found superior it must not be stopped for superiority at any stage of the trial. It is worth noting that if $B_{m_K,J} = B_{k^\star,J}$ then this corresponds to the scenario in which treatment $k^\star$ would be the last treatment dropped from the trial. Furthermore $k = \{j+1,\ldots,J\}$ in $\bigcup_{k=\{j+1,\ldots,J\}} B_{m_k,j}$ as the decision on if the trial stops for superiority at stages j<J is based on the treatment effects of the treatments left, after one of the treatments has been dropped at that stage. However the proof would still hold if one instead required all the treatments at a given stage to be superior to the control including the one that is dropped as $\bigcup_{k=\{j+1,\ldots,J\}} B_{m_k,j} \subseteq \bigcup_{k=\{j,\ldots,J\}} B_{m_k,j}$.

Therefore the type I error of treatment $k^\star$ for given $m_1, \ldots m_K$ equals $1-P(R)$ As $B_{k^\star,j} \subseteq D_{k^\star,j}$,

$$R \supseteq \bigcap_{j=1}^{J-1} \left(B_{k^\star,j} \cup \bigcup_{k=\{j+1,\ldots,J\}} B_{m_k,j} \right) \cap \left(B_{k^\star,J} \cup B_{m_K,J} \right) \supseteq \bigcap_{j=1}^{J-1} B_{k^\star,j} \cap B_{k^\star,J},$$

so

$$P(R) \geq P(\bigcap_{j=1}^{J-1} B_{k^\star,j} \cap B_{k^\star,J}),$$

therefore if $1 - P(\bigcap_{j=1}^{J} B_{k^\star,j}) \leq \alpha$ then $1 - P(R) \leq \alpha$. □

3 Equations for the proposed approach to calculate the Power under the LFC for the motivating example

To calculate the power under the LFC we calculate $P(\Phi_1)$, $P(\Phi_2)$ and $P(\Phi_3)$. As the trial has an equal number of patients per stage per arm n is used as $n = n_{1,k} = n_{2,k} - n_{1,k} = n_{3,k} - n_{2,k}$ for all $k \in \{0, 1, 2, 3\}$. $P(\Phi_1)$ equals

$$P(\Phi_1) = 2\left[\int_{u_1}^{\infty} \int_{0}^{\infty} \int_{0}^{\infty} \int_{u_1}^{\infty} \int_{0}^{\infty} \phi\left(\mathbf{z}, \mu^{\Phi_1}, \Sigma^{\Phi_1}\right) \mathbf{dz} \right],$$

where

$$\mu^{\Phi_1} = \left(\frac{\theta' \sqrt{n}}{\sigma\sqrt{2}}, \frac{(\theta' - \theta_0)\sqrt{n}}{\sigma\sqrt{2}}, \frac{(\theta' - \theta_0)\sqrt{n}}{\sigma\sqrt{2}}, \frac{\theta_0\sqrt{n}}{\sigma\sqrt{2}}, 0 \right),$$

and

$$\Sigma^{\Phi_1} = \begin{pmatrix} 1 & \frac{1}{2} & \frac{1}{2} & \frac{1}{2} & 0 \\ \frac{1}{2} & 1 & \frac{1}{2} & -\frac{1}{2} & -\frac{1}{2} \\ \frac{1}{2} & \frac{1}{2} & 1 & 0 & \frac{1}{2} \\ \frac{1}{2} & -\frac{1}{2} & 0 & 1 & \frac{1}{2} \\ 0 & -\frac{1}{2} & \frac{1}{2} & \frac{1}{2} & 1 \end{pmatrix}.$$

It is worth noting that for the events $C_{k,k^\star,j}$ one can use the fact this event equals $Z_{k,j} - Z_{k^\star,j} > 0$, to create a well-defined space to integrate over. For $P(\Phi_1)$ we are calculating

the integrals across: $Z_{1,1}, Z_{1,1} - Z_{2,1}, Z_{1,1} - Z_{3,1}, Z_{2,1}, Z_{2,1} - Z_{3,1}$. Therefore to calculate Σ^{Φ_1} one needs to find the covariance between: $Z_{1,1}$ and $Z_{1,1}$; $Z_{1,1}$ and $Z_{1,1} - Z_{2,1}$; $Z_{1,1}$ and $Z_{1,1} - Z_{3,1}$; $\ldots$; $Z_{2,1}$ and $Z_{2,1} - Z_{3,1}$; $Z_{2,1} - Z_{3,1}$ and $Z_{2,1} - Z_{3,1}$. Subsection 3.1 of the Supporting Information provides generalized equations to calculate the elements of the covariance matrix when $V_{k,j}$ can be written in the form $V_{k,j} = \sigma^2(n_{k,j} + n_{0,j})$, where $n_{k,j} = n_{k^\star,j}$ for all $k, k^\star$, as done for the motivating example. Furthermore, this formulation takes advantage of the fact that under the LFC both active treatments 2 and 3 have the same effect of interest. Thus, rather than calculating the above expression with treatment 2 being dropped first instead of treatment 3, one can simply double the integral.

$P(\Phi_2)$ equals

$$\begin{aligned}
P(\Phi_2) = 2\Bigg[&\int_{u_2}^{\infty}\int_{0}^{\infty}\int_{0}^{\infty}\int_{0}^{\infty}\int_{-\infty}^{u_1}\int_{-\infty}^{u_1} \phi\Big(\mathbf{z}, \mu^{\Phi_2}, \Sigma^{\Phi_2}\Big)\mathbf{dz} \\
&+ \int_{u_2}^{\infty}\int_{0}^{\infty}\int_{0}^{\infty}\int_{0}^{\infty}\int_{u_1}^{\infty}\int_{-\infty}^{u_1} \phi\Big(\mathbf{z}, \mu^{\Phi_2}, \Sigma^{\Phi_2}\Big)\mathbf{dz} \\
&+ \int_{u_2}^{\infty}\int_{0}^{\infty}\int_{0}^{\infty}\int_{0}^{\infty}\int_{-\infty}^{u_1}\int_{u_1}^{\infty} \phi\Big(\mathbf{z}, \mu^{\Phi_2}, \Sigma^{\Phi_2}\Big)\mathbf{dz}\Bigg],
\end{aligned}$$

where

$$\mu^{\Phi_2} = \left(\frac{\theta'\sqrt{n}}{\sigma}, \frac{(\theta' - \theta_0)\sqrt{n}}{\sigma}, \frac{(\theta' - \theta_0)\sqrt{n}}{\sigma\sqrt{2}}, 0, \frac{\theta'\sqrt{n}}{\sigma\sqrt{2}}, \frac{\theta_0\sqrt{n}}{\sigma\sqrt{2}}\right),$$

and

$$\Sigma^{\Phi_2} = \begin{pmatrix}
1 & \frac{1}{2} & \frac{1}{2\sqrt{2}} & 0 & \frac{1}{\sqrt{2}} & \frac{1}{2\sqrt{2}} \\
\frac{1}{2} & 1 & \frac{1}{2\sqrt{2}} & -\frac{1}{2\sqrt{2}} & \frac{1}{2\sqrt{2}} & -\frac{1}{2\sqrt{2}} \\
\frac{1}{2\sqrt{2}} & \frac{1}{2\sqrt{2}} & 1 & \frac{1}{2} & \frac{1}{2} & 0 \\
0 & -\frac{1}{2\sqrt{2}} & \frac{1}{2} & 1 & 0 & \frac{1}{2} \\
\frac{1}{\sqrt{2}} & \frac{1}{2\sqrt{2}} & \frac{1}{2} & 0 & 1 & \frac{1}{2} \\
\frac{1}{2\sqrt{2}} & -\frac{1}{2\sqrt{2}} & 0 & \frac{1}{2} & \frac{1}{2} & 1
\end{pmatrix}.$$

For $P(\Phi_2)$ we calculated the integrals across: $Z_{1,2}, Z_{1,2} - Z_{2,2}, Z_{1,1} - Z_{3,1}, Z_{2,1} - Z_{3,1}, Z_{1,1}, Z_{2,1}$. $P(\Phi_3)$ equals

$$\begin{aligned}
P(\Phi_3) = 2\Bigg[&\int_{u_3}^{\infty}\int_{0}^{\infty}\int_{0}^{\infty}\int_{0}^{\infty}\int_{-\infty}^{u_1}\int_{-\infty}^{u_1}\int_{-\infty}^{u_2} \phi\Big(\mathbf{z}, \mu^{\Phi_3}, \Sigma^{\Phi_3}\Big)\mathbf{dz} \\
&+ \int_{u_3}^{\infty}\int_{0}^{\infty}\int_{0}^{\infty}\int_{0}^{\infty}\int_{u_1}^{\infty}\int_{-\infty}^{u_1}\int_{-\infty}^{u_2} \phi\Big(\mathbf{z}, \mu^{\Phi_3}, \Sigma^{\Phi_3}\Big)\mathbf{dz} \\
&+ \int_{u_3}^{\infty}\int_{0}^{\infty}\int_{0}^{\infty}\int_{0}^{\infty}\int_{-\infty}^{u_1}\int_{u_1}^{\infty}\int_{-\infty}^{u_2} \phi\Big(\mathbf{z}, \mu^{\Phi_3}, \Sigma^{\Phi_3}\Big)\mathbf{dz}\Bigg],
\end{aligned}$$

where

$$\mu^{\Phi_3} = \left(\frac{\theta'\sqrt{3n}}{\sigma\sqrt{2}}, \frac{(\theta' - \theta_0)\sqrt{n}}{\sigma\sqrt{2}}, 0, \frac{(\theta' - \theta_0)\sqrt{n}}{\sigma}, \frac{\theta'\sqrt{n}}{\sigma\sqrt{2}}, \frac{\theta_0\sqrt{n}}{\sigma\sqrt{2}}, \frac{\theta'\sqrt{n}}{\sigma}\right),$$

and

$$\Sigma^{\Phi_3} = \begin{pmatrix} 1 & \frac{1}{2\sqrt{3}} & 0 & \frac{1}{\sqrt{6}} & \frac{1}{\sqrt{3}} & \frac{1}{2\sqrt{3}} & \frac{\sqrt{2}}{\sqrt{3}} \\ \frac{1}{2\sqrt{3}} & 1 & \frac{1}{2} & \frac{1}{2\sqrt{2}} & \frac{1}{2} & 0 & \frac{1}{2\sqrt{2}} \\ 0 & \frac{1}{2} & 1 & -\frac{1}{2\sqrt{2}} & 0 & \frac{1}{2} & 0 \\ \frac{1}{\sqrt{6}} & \frac{1}{2\sqrt{2}} & -\frac{1}{2\sqrt{2}} & 1 & \frac{1}{2\sqrt{2}} & -\frac{1}{2\sqrt{2}} & \frac{1}{2} \\ \frac{1}{\sqrt{3}} & \frac{1}{2} & 0 & \frac{1}{2\sqrt{2}} & 1 & \frac{1}{2} & \frac{1}{\sqrt{2}} \\ \frac{1}{2\sqrt{3}} & 0 & \frac{1}{2} & -\frac{1}{2\sqrt{2}} & \frac{1}{2} & 1 & \frac{1}{2\sqrt{2}} \\ \frac{\sqrt{2}}{\sqrt{3}} & \frac{1}{2\sqrt{2}} & 0 & \frac{1}{2} & \frac{1}{\sqrt{2}} & \frac{1}{2\sqrt{2}} & 1 \end{pmatrix}.$$

For $P(\Phi_3)$ we calculated the integrals across: $Z_{1,3}$, $Z_{1,1}-Z_{3,1}$, $Z_{2,1}-Z_{3,1}$, $Z_{1,2}-Z_{2,2}$, $Z_{1,1}$, $Z_{2,1}$, $Z_{1,2}$. The power under the LFC for the motivating example is therefore

$$\sum_{j=1}^{3} P(\Phi_j).$$

3.1 General equation for covariance matrix

Under the same assumptions as used in Wason et al. (2017) of $V_{k,j} = \sigma^2(n_{k,j}^{-1} + n_{0,j}^{-1})$ where $n_{k,j} = n_{k^\star,j}$ for all $k, k^\star$ the covariance between the events $B_{k,j}$ and $B_{k^\star,j^\star}$; or $B_{k,j}$ and $A_{k^\star,j^\star}$; or $A_{k,j}$ and $B_{k^\star,j^\star}$; or $A_{k,j}$ and $A_{k^\star,j^\star}$ equals:

$$\text{Cov}(Z_{k,j}, Z_{k^\star,j^\star}) = \begin{cases} \sqrt{\frac{\min(n_j,n_{j^\star})}{\max(n_j,n_{j^\star})}} & \text{if } k^\star = k \\ \frac{1}{2}\sqrt{\frac{\min(n_j,n_{j^\star})}{\max(n_j,n_{j^\star})}} & \text{if } k^\star \neq k \end{cases}.$$

The covariance between the events $C_{k_1,k_2,j}$ and $C_{k_1^\star,k_2^\star,j^\star}$ equals:

$$\text{Cov}(Z_{k_1,j}-Z_{k_2,j}, Z_{k_1^\star,j^\star}-Z_{k_2^\star,j^\star}) = \begin{cases} \sqrt{\frac{\min(n_j,n_{j^\star})}{\max(n_j,n_{j^\star})}} & \text{if } k_1^\star = k_1 \;\&\; k_2^\star = k_2 \\ -\sqrt{\frac{\min(n_j,n_{j^\star})}{\max(n_j,n_{j^\star})}} & \text{if } k_2^\star = k_1 \;\&\; k_1^\star = k_2 \\ \frac{1}{2}\sqrt{\frac{\min(n_j,n_{j^\star})}{\max(n_j,n_{j^\star})}} & \text{if } k_1^\star = k_1 \;\&\; k_2^\star \neq k_2 \text{ or } k_1^\star \neq k_1 \;\&\; k_2^\star = k_2 \\ -\frac{1}{2}\sqrt{\frac{\min(n_j,n_{j^\star})}{\max(n_j,n_{j^\star})}} & \text{if } k_1^\star = k_2 \;\&\; k_2^\star \neq k_1 \text{ or } k_1^\star \neq k_2 \;\&\; k_2^\star = k_1 \\ 0 & \text{if } k_1^\star \neq k_1 \;\&\; k_1^\star \neq k_2 \;\&\; k_2^\star \neq k_1 \;\&\; k_2^\star \neq k_2 \end{cases}.$$

It is worth noting that by design $k_1 \neq k_2$ and $k_1^\star \neq k_2^\star$. The covariance between the events $A_{k_1,j}$ and $C_{k_1^\star,k_2^\star,j^\star}$; or $B_{k_1,j}$ and $C_{k_1^\star,k_2^\star,j^\star}$ equals:

$$\text{Cov}(Z_{k_1,j}, Z_{k_1^\star,j^\star} - Z_{k_2^\star,j^\star}) = \begin{cases} \frac{1}{2}\sqrt{\frac{\min(n_j,n_{j^\star})}{\max(n_j,n_{j^\star})}} & \text{if } k_1 = k_1^\star \\ -\frac{1}{2}\sqrt{\frac{\min(n_j,n_{j^\star})}{\max(n_j,n_{j^\star})}} & \text{if } k_1 = k_2^\star \\ 0 & \text{if } k_1 \neq k_1^\star \;\&\; k_1 \neq k_2^\star \end{cases}.$$

4 Equations for the proposed approach to calculate the expected sample size for the motivating example

To calculate the expected sample size for the given Δ we calculate $P(\Psi_1)$, $P(\Psi_2)$ and $P(\Psi_3)$. $P(\Psi_1)$ equals

$$P(\Psi_1) = \int_{u_1}^{\infty}\int_{0}^{\infty}\int_{u_1}^{\infty}\int_{0}^{\infty} \phi\Big(\mathbf{z}, \mu^{\Psi_{1,a}}, \Sigma^{\Psi_1}\Big)\mathbf{dz} + \int_{u_1}^{\infty}\int_{0}^{\infty}\int_{u_1}^{\infty}\int_{0}^{\infty} \phi\Big(\mathbf{z}, \mu^{\Psi_{1,b}}, \Sigma^{\Psi_1}\Big)\mathbf{dz}$$
$$+ \int_{u_1}^{\infty}\int_{0}^{\infty}\int_{u_1}^{\infty}\int_{0}^{\infty} \phi\Big(\mathbf{z}, \mu^{\Psi_{1,c}}, \Sigma^{\Psi_1}\Big)\mathbf{dz},$$

where

$$\mu^{\Psi_{1,a}} = \left(\frac{\delta_{i_1}\sqrt{n}}{\sigma\sqrt{2}}, \frac{(\delta_{i_1}-\delta_{i_3})\sqrt{n}}{\sigma\sqrt{2}}, \frac{\delta_{i_2}\sqrt{n}}{\sigma\sqrt{2}}, \frac{(\delta_{i_2}-\delta_{i_3})\sqrt{n}}{\sigma\sqrt{2}}\right),$$

with $i_1 = 1, i_2 = 2, i_3 = 3$;

$$\mu^{\Psi_{1,b}} = \left(\frac{\delta_{i_1}\sqrt{n}}{\sigma\sqrt{2}}, \frac{(\delta_{i_1}-\delta_{i_3})\sqrt{n}}{\sigma\sqrt{2}}, \frac{\delta_{i_2}\sqrt{n}}{\sigma\sqrt{2}}, \frac{(\delta_{i_2}-\delta_{i_3})\sqrt{n}}{\sigma\sqrt{2}}\right),$$

with $i_1 = 1, i_2 = 3, i_3 = 2$;

$$\mu^{\Psi_{1,c}} = \left(\frac{\delta_{i_1}\sqrt{n}}{\sigma\sqrt{2}}, \frac{(\delta_{i_1}-\delta_{i_3})\sqrt{n}}{\sigma\sqrt{2}}, \frac{\delta_{i_2}\sqrt{n}}{\sigma\sqrt{2}}, \frac{(\delta_{i_2}-\delta_{i_3})\sqrt{n}}{\sigma\sqrt{2}}\right),$$

with $i_1 = 2, i_2 = 3, i_3 = 1$ and

$$\Sigma^{\Psi_1} = \begin{pmatrix} 1 & \frac{1}{2} & \frac{1}{2} & 0 \\ \frac{1}{2} & 1 & 0 & \frac{1}{2} \\ \frac{1}{2} & 0 & 1 & \frac{1}{2} \\ 0 & \frac{1}{2} & \frac{1}{2} & 1 \end{pmatrix}.$$

$P(\Psi_2)$ equals

$$
\begin{aligned}
P(\Psi_2) = &\int_{u_2}^{\infty}\int_{0}^{\infty}\int_{0}^{\infty}\int_{0}^{\infty}\int_{-\infty}^{u_1}\int_{-\infty}^{u_1}\phi\Big(\mathbf{z},\mu^{\Psi_{2,a}},\Sigma^{\Psi_2}\Big)\mathbf{dz}\\
&+\int_{u_2}^{\infty}\int_{0}^{\infty}\int_{0}^{\infty}\int_{0}^{\infty}\int_{u_1}^{\infty}\int_{-\infty}^{u_1}\phi\Big(\mathbf{z},\mu^{\Psi_{2,a}},\Sigma^{\Psi_2}\Big)\mathbf{dz}\\
&+\int_{u_2}^{\infty}\int_{0}^{\infty}\int_{0}^{\infty}\int_{0}^{\infty}\int_{-\infty}^{u_1}\int_{u_1}^{\infty}\phi\Big(\mathbf{z},\mu^{\Psi_{2,a}},\Sigma^{\Psi_2}\Big)\mathbf{dz}\\
&+\int_{u_2}^{\infty}\int_{0}^{\infty}\int_{0}^{\infty}\int_{0}^{\infty}\int_{-\infty}^{u_1}\int_{-\infty}^{u_1}\phi\Big(\mathbf{z},\mu^{\Psi_{2,b}},\Sigma^{\Psi_2}\Big)\mathbf{dz}\\
&+\int_{u_2}^{\infty}\int_{0}^{\infty}\int_{0}^{\infty}\int_{0}^{\infty}\int_{u_1}^{\infty}\int_{-\infty}^{u_1}\phi\Big(\mathbf{z},\mu^{\Psi_{2,b}},\Sigma^{\Psi_2}\Big)\mathbf{dz}\\
&+\int_{u_2}^{\infty}\int_{0}^{\infty}\int_{0}^{\infty}\int_{0}^{\infty}\int_{-\infty}^{u_1}\int_{u_1}^{\infty}\phi\Big(\mathbf{z},\mu^{\Psi_{2,b}},\Sigma^{\Psi_2}\Big)\mathbf{dz}\\
&+\int_{u_2}^{\infty}\int_{0}^{\infty}\int_{0}^{\infty}\int_{0}^{\infty}\int_{-\infty}^{u_1}\int_{-\infty}^{u_1}\phi\Big(\mathbf{z},\mu^{\Psi_{2,c}},\Sigma^{\Psi_2}\Big)\mathbf{dz}\\
&+\int_{u_2}^{\infty}\int_{0}^{\infty}\int_{0}^{\infty}\int_{0}^{\infty}\int_{u_1}^{\infty}\int_{-\infty}^{u_1}\phi\Big(\mathbf{z},\mu^{\Psi_{2,c}},\Sigma^{\Psi_2}\Big)\mathbf{dz}\\
&+\int_{u_2}^{\infty}\int_{0}^{\infty}\int_{0}^{\infty}\int_{0}^{\infty}\int_{-\infty}^{u_1}\int_{u_1}^{\infty}\phi\Big(\mathbf{z},\mu^{\Psi_{2,c}},\Sigma^{\Psi_2}\Big)\mathbf{dz}\\
&+\int_{u_2}^{\infty}\int_{0}^{\infty}\int_{0}^{\infty}\int_{0}^{\infty}\int_{-\infty}^{u_1}\int_{-\infty}^{u_1}\phi\Big(\mathbf{z},\mu^{\Psi_{2,d}},\Sigma^{\Psi_2}\Big)\mathbf{dz}\\
&+\int_{u_2}^{\infty}\int_{0}^{\infty}\int_{0}^{\infty}\int_{0}^{\infty}\int_{u_1}^{\infty}\int_{-\infty}^{u_1}\phi\Big(\mathbf{z},\mu^{\Psi_{2,d}},\Sigma^{\Psi_2}\Big)\mathbf{dz}\\
&+\int_{u_2}^{\infty}\int_{0}^{\infty}\int_{0}^{\infty}\int_{0}^{\infty}\int_{-\infty}^{u_1}\int_{u_1}^{\infty}\phi\Big(\mathbf{z},\mu^{\Psi_{2,d}},\Sigma^{\Psi_2}\Big)\mathbf{dz}\\
&+\int_{u_2}^{\infty}\int_{0}^{\infty}\int_{0}^{\infty}\int_{0}^{\infty}\int_{-\infty}^{u_1}\int_{-\infty}^{u_1}\phi\Big(\mathbf{z},\mu^{\Psi_{2,e}},\Sigma^{\Psi_2}\Big)\mathbf{dz}\\
&+\int_{u_2}^{\infty}\int_{0}^{\infty}\int_{0}^{\infty}\int_{0}^{\infty}\int_{u_1}^{\infty}\int_{-\infty}^{u_1}\phi\Big(\mathbf{z},\mu^{\Psi_{2,e}},\Sigma^{\Psi_2}\Big)\mathbf{dz}\\
&+\int_{u_2}^{\infty}\int_{0}^{\infty}\int_{0}^{\infty}\int_{0}^{\infty}\int_{-\infty}^{u_1}\int_{u_1}^{\infty}\phi\Big(\mathbf{z},\mu^{\Psi_{2,e}},\Sigma^{\Psi_2}\Big)\mathbf{dz}\\
&+\int_{u_2}^{\infty}\int_{0}^{\infty}\int_{0}^{\infty}\int_{0}^{\infty}\int_{-\infty}^{u_1}\int_{-\infty}^{u_1}\phi\Big(\mathbf{z},\mu^{\Psi_{2,f}},\Sigma^{\Psi_2}\Big)\mathbf{dz}\\
&+\int_{u_2}^{\infty}\int_{0}^{\infty}\int_{0}^{\infty}\int_{0}^{\infty}\int_{u_1}^{\infty}\int_{-\infty}^{u_1}\phi\Big(\mathbf{z},\mu^{\Psi_{2,f}},\Sigma^{\Psi_2}\Big)\mathbf{dz}\\
&+\int_{u_2}^{\infty}\int_{0}^{\infty}\int_{0}^{\infty}\int_{0}^{\infty}\int_{-\infty}^{u_1}\int_{u_1}^{\infty}\phi\Big(\mathbf{z},\mu^{\Psi_{2,f}},\Sigma^{\Psi_2}\Big)\mathbf{dz},
\end{aligned}
$$

where

$$\mu^{\Psi_{2,a}} = \left(\frac{\delta_{i_1}\sqrt{n}}{\sigma}, \frac{(\delta_{i_1} - \delta_{i_2})\sqrt{n}}{\sigma}, \frac{(\delta_{i_1} - \delta_{i_3})\sqrt{n}}{\sigma\sqrt{2}}, \frac{(\delta_{i_2} - \delta_{i_3})\sqrt{n}}{\sigma\sqrt{2}}, \frac{\delta_{i_1}\sqrt{n}}{\sigma\sqrt{2}}, \frac{\delta_{i_2}\sqrt{n}}{\sigma\sqrt{2}} \right),$$

with $i_1 = 1, i_2 = 2, i_3 = 3$;

$$\mu^{\Psi_{2,b}} = \left(\frac{\delta_{i_1}\sqrt{n}}{\sigma}, \frac{(\delta_{i_1} - \delta_{i_2})\sqrt{n}}{\sigma}, \frac{(\delta_{i_1} - \delta_{i_3})\sqrt{n}}{\sigma\sqrt{2}}, \frac{(\delta_{i_2} - \delta_{i_3})\sqrt{n}}{\sigma\sqrt{2}}, \frac{\delta_{i_1}\sqrt{n}}{\sigma\sqrt{2}}, \frac{\delta_{i_2}\sqrt{n}}{\sigma\sqrt{2}} \right),$$

with $i_1 = 1, i_2 = 3, i_3 = 2$;

$$\mu^{\Psi_{2,c}} = \left(\frac{\delta_{i_1}\sqrt{n}}{\sigma}, \frac{(\delta_{i_1} - \delta_{i_2})\sqrt{n}}{\sigma}, \frac{(\delta_{i_1} - \delta_{i_3})\sqrt{n}}{\sigma\sqrt{2}}, \frac{(\delta_{i_2} - \delta_{i_3})\sqrt{n}}{\sigma\sqrt{2}}, \frac{\delta_{i_1}\sqrt{n}}{\sigma\sqrt{2}}, \frac{\delta_{i_2}\sqrt{n}}{\sigma\sqrt{2}} \right),$$

with $i_1 = 2, i_2 = 3, i_3 = 1$;

$$\mu^{\Psi_{2,d}} = \left(\frac{\delta_{i_1}\sqrt{n}}{\sigma}, \frac{(\delta_{i_1} - \delta_{i_2})\sqrt{n}}{\sigma}, \frac{(\delta_{i_1} - \delta_{i_3})\sqrt{n}}{\sigma\sqrt{2}}, \frac{(\delta_{i_2} - \delta_{i_3})\sqrt{n}}{\sigma\sqrt{2}}, \frac{\delta_{i_1}\sqrt{n}}{\sigma\sqrt{2}}, \frac{\delta_{i_2}\sqrt{n}}{\sigma\sqrt{2}} \right),$$

with $i_1 = 3, i_2 = 2, i_3 = 1$;

$$\mu^{\Psi_{2,e}} = \left(\frac{\delta_{i_1}\sqrt{n}}{\sigma}, \frac{(\delta_{i_1} - \delta_{i_2})\sqrt{n}}{\sigma}, \frac{(\delta_{i_1} - \delta_{i_3})\sqrt{n}}{\sigma\sqrt{2}}, \frac{(\delta_{i_2} - \delta_{i_3})\sqrt{n}}{\sigma\sqrt{2}}, \frac{\delta_{i_1}\sqrt{n}}{\sigma\sqrt{2}}, \frac{\delta_{i_2}\sqrt{n}}{\sigma\sqrt{2}} \right),$$

with $i_1 = 3, i_2 = 1, i_3 = 2$;

$$\mu^{\Psi_{2,f}} = \left(\frac{\delta_{i_1}\sqrt{n}}{\sigma}, \frac{(\delta_{i_1} - \delta_{i_2})\sqrt{n}}{\sigma}, \frac{(\delta_{i_1} - \delta_{i_3})\sqrt{n}}{\sigma\sqrt{2}}, \frac{(\delta_{i_2} - \delta_{i_3})\sqrt{n}}{\sigma\sqrt{2}}, \frac{\delta_{i_1}\sqrt{n}}{\sigma\sqrt{2}}, \frac{\delta_{i_2}\sqrt{n}}{\sigma\sqrt{2}} \right),$$

with $i_1 = 2, i_2 = 1, i_3 = 3$ and

$$\Sigma^{\Psi_2} = \begin{pmatrix} 1 & \frac{1}{2} & \frac{1}{2\sqrt{2}} & 0 & \frac{1}{\sqrt{2}} & \frac{1}{2\sqrt{2}} \\ \frac{1}{2} & 1 & \frac{1}{2\sqrt{2}} & -\frac{1}{2\sqrt{2}} & \frac{1}{2\sqrt{2}} & -\frac{1}{2\sqrt{2}} \\ \frac{1}{2\sqrt{2}} & \frac{1}{2\sqrt{2}} & 1 & \frac{1}{2} & \frac{1}{2} & 0 \\ 0 & -\frac{1}{2\sqrt{2}} & \frac{1}{2} & 1 & 0 & \frac{1}{2} \\ \frac{1}{\sqrt{2}} & \frac{1}{2\sqrt{2}} & \frac{1}{2} & 0 & 1 & \frac{1}{2} \\ \frac{1}{2\sqrt{2}} & -\frac{1}{2\sqrt{2}} & 0 & \frac{1}{2} & \frac{1}{2} & 1 \end{pmatrix}.$$

$P(\Psi_3)$ equals

$$
\begin{aligned}
P(\Psi_3) = & \int_0^\infty \int_0^\infty \int_0^\infty \int_{-\infty}^{u_1} \int_{-\infty}^{u_1} \int_{-\infty}^{u_2} \phi\Big(\mathbf{z}, \mu^{\Psi_{3,a}}, \Sigma^{\Psi_3}\Big)\mathbf{dz} \\
& + \int_0^\infty \int_0^\infty \int_0^\infty \int_{u_1}^{\infty} \int_{-\infty}^{u_1} \int_{-\infty}^{u_2} \phi\Big(\mathbf{z}, \mu^{\Psi_{3,a}}, \Sigma^{\Psi_3}\Big)\mathbf{dz} \\
& + \int_0^\infty \int_0^\infty \int_0^\infty \int_{-\infty}^{u_1} \int_{u_1}^{\infty} \int_{-\infty}^{u_2} \phi\Big(\mathbf{z}, \mu^{\Psi_{3,a}}, \Sigma^{\Psi_3}\Big)\mathbf{dz} \\
& + \int_0^\infty \int_0^\infty \int_0^\infty \int_{-\infty}^{u_1} \int_{-\infty}^{u_1} \int_{-\infty}^{u_2} \phi\Big(\mathbf{z}, \mu^{\Psi_{3,b}}, \Sigma^{\Psi_3}\Big)\mathbf{dz} \\
& + \int_0^\infty \int_0^\infty \int_0^\infty \int_{u_1}^{\infty} \int_{-\infty}^{u_1} \int_{-\infty}^{u_2} \phi\Big(\mathbf{z}, \mu^{\Psi_{3,b}}, \Sigma^{\Psi_3}\Big)\mathbf{dz} \\
& + \int_0^\infty \int_0^\infty \int_0^\infty \int_{-\infty}^{u_1} \int_{u_1}^{\infty} \int_{-\infty}^{u_2} \phi\Big(\mathbf{z}, \mu^{\Psi_{3,b}}, \Sigma^{\Psi_3}\Big)\mathbf{dz} \\
& + \int_0^\infty \int_0^\infty \int_0^\infty \int_{-\infty}^{u_1} \int_{-\infty}^{u_1} \int_{-\infty}^{u_2} \phi\Big(\mathbf{z}, \mu^{\Psi_{3,c}}, \Sigma^{\Psi_3}\Big)\mathbf{dz} \\
& + \int_0^\infty \int_0^\infty \int_0^\infty \int_{u_1}^{\infty} \int_{-\infty}^{u_1} \int_{-\infty}^{u_2} \phi\Big(\mathbf{z}, \mu^{\Psi_{3,c}}, \Sigma^{\Psi_3}\Big)\mathbf{dz} \\
& + \int_0^\infty \int_0^\infty \int_0^\infty \int_{-\infty}^{u_1} \int_{u_1}^{\infty} \int_{-\infty}^{u_2} \phi\Big(\mathbf{z}, \mu^{\Psi_{3,c}}, \Sigma^{\Psi_3}\Big)\mathbf{dz} \\
& + \int_0^\infty \int_0^\infty \int_0^\infty \int_{-\infty}^{u_1} \int_{-\infty}^{u_1} \int_{-\infty}^{u_2} \phi\Big(\mathbf{z}, \mu^{\Psi_{3,d}}, \Sigma^{\Psi_3}\Big)\mathbf{dz} \\
& + \int_0^\infty \int_0^\infty \int_0^\infty \int_{u_1}^{\infty} \int_{-\infty}^{u_1} \int_{-\infty}^{u_2} \phi\Big(\mathbf{z}, \mu^{\Psi_{3,d}}, \Sigma^{\Psi_3}\Big)\mathbf{dz} \\
& + \int_0^\infty \int_0^\infty \int_0^\infty \int_{-\infty}^{u_1} \int_{u_1}^{\infty} \int_{-\infty}^{u_2} \phi\Big(\mathbf{z}, \mu^{\Psi_{3,d}}, \Sigma^{\Psi_3}\Big)\mathbf{dz} \\
& + \int_0^\infty \int_0^\infty \int_0^\infty \int_{-\infty}^{u_1} \int_{-\infty}^{u_1} \int_{-\infty}^{u_2} \phi\Big(\mathbf{z}, \mu^{\Psi_{3,e}}, \Sigma^{\Psi_3}\Big)\mathbf{dz} \\
& + \int_0^\infty \int_0^\infty \int_0^\infty \int_{u_1}^{\infty} \int_{-\infty}^{u_1} \int_{-\infty}^{u_2} \phi\Big(\mathbf{z}, \mu^{\Psi_{3,e}}, \Sigma^{\Psi_3}\Big)\mathbf{dz} \\
& + \int_0^\infty \int_0^\infty \int_0^\infty \int_{-\infty}^{u_1} \int_{u_1}^{\infty} \int_{-\infty}^{u_2} \phi\Big(\mathbf{z}, \mu^{\Psi_{3,e}}, \Sigma^{\Psi_3}\Big)\mathbf{dz} \\
& + \int_0^\infty \int_0^\infty \int_0^\infty \int_{-\infty}^{u_1} \int_{-\infty}^{u_1} \int_{-\infty}^{u_2} \phi\Big(\mathbf{z}, \mu^{\Psi_{3,f}}, \Sigma^{\Psi_3}\Big)\mathbf{dz} \\
& + \int_0^\infty \int_0^\infty \int_0^\infty \int_{u_1}^{\infty} \int_{-\infty}^{u_1} \int_{-\infty}^{u_2} \phi\Big(\mathbf{z}, \mu^{\Psi_{3,f}}, \Sigma^{\Psi_3}\Big)\mathbf{dz} \\
& + \int_0^\infty \int_0^\infty \int_0^\infty \int_{-\infty}^{u_1} \int_{u_1}^{\infty} \int_{-\infty}^{u_2} \phi\Big(\mathbf{z}, \mu^{\Psi_{3,f}}, \Sigma^{\Psi_3}\Big)\mathbf{dz},
\end{aligned}
$$

where

$$\mu^{\Psi_{3,a}} = \left(\frac{(\delta_{i_1} - \delta_{i_3})\sqrt{n}}{\sigma\sqrt{2}}, \frac{(\delta_{i_2} - \delta_{i_3})\sqrt{n}}{\sigma\sqrt{2}}, \frac{(\delta_{i_1} - \delta_{i_2})\sqrt{n}}{\sigma}, \frac{\delta_{i_1}\sqrt{n}}{\sigma\sqrt{2}}, \frac{\delta_{i_2}\sqrt{n}}{\sigma\sqrt{2}}, \frac{\delta_{i_1}\sqrt{n}}{\sigma}\right),$$

with $i_1 = 1, i_2 = 2, i_3 = 3$;

$$\mu^{\Psi_{3,b}} = \left(\frac{(\delta_{i_1} - \delta_{i_3})\sqrt{n}}{\sigma\sqrt{2}}, \frac{(\delta_{i_2} - \delta_{i_3})\sqrt{n}}{\sigma\sqrt{2}}, \frac{(\delta_{i_1} - \delta_{i_2})\sqrt{n}}{\sigma}, \frac{\delta_{i_1}\sqrt{n}}{\sigma\sqrt{2}}, \frac{\delta_{i_2}\sqrt{n}}{\sigma\sqrt{2}}, \frac{\delta_{i_1}\sqrt{n}}{\sigma}\right),$$

with $i_1 = 1, i_2 = 3, i_3 = 2$;

$$\mu^{\Psi_{3,c}} = \left(\frac{(\delta_{i_1} - \delta_{i_3})\sqrt{n}}{\sigma\sqrt{2}}, \frac{(\delta_{i_2} - \delta_{i_3})\sqrt{n}}{\sigma\sqrt{2}}, \frac{(\delta_{i_1} - \delta_{i_2})\sqrt{n}}{\sigma}, \frac{\delta_{i_1}\sqrt{n}}{\sigma\sqrt{2}}, \frac{\delta_{i_2}\sqrt{n}}{\sigma\sqrt{2}}, \frac{\delta_{i_1}\sqrt{n}}{\sigma}\right),$$

with $i_1 = 2, i_2 = 3, i_3 = 1$;

$$\mu^{\Psi_{3,d}} = \left(\frac{(\delta_{i_1} - \delta_{i_3})\sqrt{n}}{\sigma\sqrt{2}}, \frac{(\delta_{i_2} - \delta_{i_3})\sqrt{n}}{\sigma\sqrt{2}}, \frac{(\delta_{i_1} - \delta_{i_2})\sqrt{n}}{\sigma}, \frac{\delta_{i_1}\sqrt{n}}{\sigma\sqrt{2}}, \frac{\delta_{i_2}\sqrt{n}}{\sigma\sqrt{2}}, \frac{\delta_{i_1}\sqrt{n}}{\sigma}\right),$$

with $i_1 = 3, i_2 = 2, i_3 = 1$;

$$\mu^{\Psi_{3,e}} = \left(\frac{(\delta_{i_1} - \delta_{i_3})\sqrt{n}}{\sigma\sqrt{2}}, \frac{(\delta_{i_2} - \delta_{i_3})\sqrt{n}}{\sigma\sqrt{2}}, \frac{(\delta_{i_1} - \delta_{i_2})\sqrt{n}}{\sigma}, \frac{\delta_{i_1}\sqrt{n}}{\sigma\sqrt{2}}, \frac{\delta_{i_2}\sqrt{n}}{\sigma\sqrt{2}}, \frac{\delta_{i_1}\sqrt{n}}{\sigma}\right),$$

with $i_1 = 3, i_2 = 1, i_3 = 2$;

$$\mu^{\Psi_{3,f}} = \left(\frac{(\delta_{i_1} - \delta_{i_3})\sqrt{n}}{\sigma\sqrt{2}}, \frac{(\delta_{i_2} - \delta_{i_3})\sqrt{n}}{\sigma\sqrt{2}}, \frac{(\delta_{i_1} - \delta_{i_2})\sqrt{n}}{\sigma}, \frac{\delta_{i_1}\sqrt{n}}{\sigma\sqrt{2}}, \frac{\delta_{i_2}\sqrt{n}}{\sigma\sqrt{2}}, \frac{\delta_{i_1}\sqrt{n}}{\sigma}\right),$$

with $i_1 = 2, i_2 = 1, i_3 = 3$ and

$$\Sigma^{\Psi_3} = \begin{pmatrix} 1 & \frac{1}{2} & \frac{1}{2\sqrt{2}} & \frac{1}{2} & 0 & \frac{1}{2\sqrt{2}} \\ \frac{1}{2} & 1 & -\frac{1}{2\sqrt{2}} & 0 & \frac{1}{2} & 0 \\ \frac{1}{2\sqrt{2}} & -\frac{1}{2\sqrt{2}} & 1 & \frac{1}{2\sqrt{2}} & -\frac{1}{2\sqrt{2}} & \frac{1}{2} \\ \frac{1}{2} & 0 & \frac{1}{2\sqrt{2}} & 1 & \frac{1}{2} & \frac{1}{\sqrt{2}} \\ 0 & \frac{1}{2} & -\frac{1}{2\sqrt{2}} & \frac{1}{2} & 1 & \frac{1}{2\sqrt{2}} \\ \frac{1}{2\sqrt{2}} & 0 & \frac{1}{2} & \frac{1}{\sqrt{2}} & \frac{1}{2\sqrt{2}} & 1 \end{pmatrix}.$$

The expected sample size is therefore

$$E(N|\Delta) = \sum_{j=1}^{3} \left(P(\Psi_j) \left(\sum_{i=1}^{j-1} in + (K - j + 2)jn \right) \right).$$

5 Equations for the proposed approach to calculate the type I error under the global null

To calculate the type I error under the global null we calculate the probability that, without loss of generality, treatment 1 is found superior to the control at a given stage j and the trial stops at that given stage j. We define this event as ν_j. This is therefore similar to Φ_1, Φ_2, Φ_3. $P(\nu_1)$ equals

$$P(\nu_1) = 2\left[\int_{u_1}^{\infty}\int_{0}^{\infty}\int_{u_1}^{\infty}\int_{0}^{\infty}\phi\left(\mathbf{z}, \mu^{\nu_{1,a}}, \Sigma^{\nu_{1,a}}\right)\mathbf{dz}\right] + \int_{u_1}^{\infty}\int_{-\infty}^{0}\int_{-\infty}^{0}\int_{u_1}^{\infty}\int_{u_1}^{\infty}\phi\left(\mathbf{z}, \mu^{\nu_{1,b}}, \Sigma^{\nu_{1,b}}\right)\mathbf{dz},$$

where

$$\begin{aligned}\mu^{\nu_{1,a}} &= \left(0, 0, 0, 0\right),\\ \mu^{\nu_{1,b}} &= \left(0, 0, 0, 0, 0\right),\end{aligned}$$

and

$$\Sigma^{\nu_{1,a}} = \begin{pmatrix} 1 & \frac{1}{2} & \frac{1}{2} & 0 \\ \frac{1}{2} & 1 & 0 & \frac{1}{2} \\ \frac{1}{2} & 0 & 1 & \frac{1}{2} \\ 0 & \frac{1}{2} & \frac{1}{2} & 1 \end{pmatrix}.$$

$$\Sigma^{\nu_{1,b}} = \begin{pmatrix} 1 & \frac{1}{2} & \frac{1}{2} & \frac{1}{2} & \frac{1}{2} \\ \frac{1}{2} & 1 & \frac{1}{2} & -\frac{1}{2} & 0 \\ \frac{1}{2} & \frac{1}{2} & 1 & 0 & -\frac{1}{2} \\ \frac{1}{2} & -\frac{1}{2} & 0 & 1 & \frac{1}{2} \\ \frac{1}{2} & 0 & -\frac{1}{2} & \frac{1}{2} & 1 \end{pmatrix}.$$

$P(\nu_2)$ equals

$$\begin{aligned}P(\nu_2) = 2\Bigg[&\int_{u_2}^{\infty}\int_{0}^{\infty}\int_{0}^{\infty}\int_{-\infty}^{u_1}\int_{-\infty}^{u_1}\phi\left(\mathbf{z}, \mu^{\nu_2}, \Sigma^{\nu_2}\right)\mathbf{dz}\\ &+\int_{u_2}^{\infty}\int_{0}^{\infty}\int_{0}^{\infty}\int_{u_1}^{\infty}\int_{-\infty}^{u_1}\phi\left(\mathbf{z}, \mu^{\nu_2}, \Sigma^{\nu_2}\right)\mathbf{dz}\\ &+\int_{u_2}^{\infty}\int_{0}^{\infty}\int_{0}^{\infty}\int_{-\infty}^{u_1}\int_{u_1}^{\infty}\phi\left(\mathbf{z}, \mu^{\nu_2}, \Sigma^{\nu_2}\right)\mathbf{dz}\Bigg],\end{aligned}$$

where

$$\mu^{\nu_2} = \left(0, 0, 0, 0, 0\right),$$

and

$$\Sigma^{\nu_2} = \begin{pmatrix} 1 & \frac{1}{2\sqrt{2}} & 0 & \frac{1}{\sqrt{2}} & \frac{1}{2\sqrt{2}} \\ \frac{1}{2\sqrt{2}} & 1 & \frac{1}{2} & \frac{1}{2} & 0 \\ 0 & \frac{1}{2} & 1 & 0 & \frac{1}{2} \\ \frac{1}{\sqrt{2}} & \frac{1}{2} & 0 & 1 & \frac{1}{2} \\ \frac{1}{2\sqrt{2}} & 0 & \frac{1}{2} & \frac{1}{2} & 1 \end{pmatrix}.$$

$P(\nu_3)$ equals

$$\begin{aligned} P(\nu_3) = 2\Bigg[& \int_{u_3}^{\infty}\int_{0}^{\infty}\int_{0}^{\infty}\int_{0}^{\infty}\int_{-\infty}^{u_1}\int_{-\infty}^{u_1}\int_{-\infty}^{u_2} \phi\Big(\mathbf{z}, \mu^{\nu_3}, \Sigma^{\nu_3}\Big)\mathbf{dz} \\ & + \int_{u_3}^{\infty}\int_{0}^{\infty}\int_{0}^{\infty}\int_{0}^{\infty}\int_{u_1}^{\infty}\int_{-\infty}^{u_1}\int_{-\infty}^{u_2} \phi\Big(\mathbf{z}, \mu^{\nu_3}, \Sigma^{\nu_3}\Big)\mathbf{dz} \\ & + \int_{u_3}^{\infty}\int_{0}^{\infty}\int_{0}^{\infty}\int_{0}^{\infty}\int_{-\infty}^{u_1}\int_{u_1}^{\infty}\int_{-\infty}^{u_2} \phi\Big(\mathbf{z}, \mu^{\nu_3}, \Sigma^{\nu_3}\Big)\mathbf{dz}\Bigg], \end{aligned}$$

where

$$\mu^{\nu_3} = \Big(0, 0, 0, 0, 0, 0, 0\Big),$$

and

$$\Sigma^{\nu_3} = \begin{pmatrix} 1 & \frac{1}{2\sqrt{3}} & 0 & \frac{1}{\sqrt{6}} & \frac{1}{\sqrt{3}} & \frac{1}{2\sqrt{3}} & \frac{\sqrt{2}}{\sqrt{3}} \\ \frac{1}{2\sqrt{3}} & 1 & \frac{1}{2} & \frac{1}{2\sqrt{2}} & \frac{1}{2} & 0 & \frac{1}{2\sqrt{2}} \\ 0 & \frac{1}{2} & 1 & -\frac{1}{2\sqrt{2}} & 0 & \frac{1}{2} & 0 \\ \frac{1}{\sqrt{6}} & \frac{1}{2\sqrt{2}} & -\frac{1}{2\sqrt{2}} & 1 & \frac{1}{2\sqrt{2}} & -\frac{1}{2\sqrt{2}} & \frac{1}{2} \\ \frac{1}{\sqrt{3}} & \frac{1}{2} & 0 & \frac{1}{2\sqrt{2}} & 1 & \frac{1}{2} & \frac{1}{\sqrt{2}} \\ \frac{1}{2\sqrt{3}} & 0 & \frac{1}{2} & -\frac{1}{2\sqrt{2}} & \frac{1}{2} & 1 & \frac{1}{2\sqrt{2}} \\ \frac{\sqrt{2}}{\sqrt{3}} & \frac{1}{2\sqrt{2}} & 0 & \frac{1}{2} & \frac{1}{\sqrt{2}} & \frac{1}{2\sqrt{2}} & 1 \end{pmatrix}.$$

The type I error for a given treatment under the global null for the motivating example is

$$\sum_{j=1}^{3} P(\nu_j).$$

6 Simulations of type I error

Figure 1 gives the type I error for treatment 1 under multiple different values of θ_1, θ_2 and θ_3. Values tested for θ_2, θ_3 are in the range of $-2\theta'$ to $2\theta'$. Values tested for θ_1 are in the range of $-\theta'$ to 0. 1,000,000 simulations of each scenario are run. The maximum value for type I error for treatment 1 is 0.02496 which is when $\theta_1 = 0$, $\theta_2 = -2\theta'$ and $\theta_3 = -2\theta'$.

References

Wason, J., Stallard, N., Bowden, J., and Jennison, C. (2017). A multi-stage drop-the-losers design for multi-arm clinical trials. *Statistical methods in medical research*, 26(1):508–524.

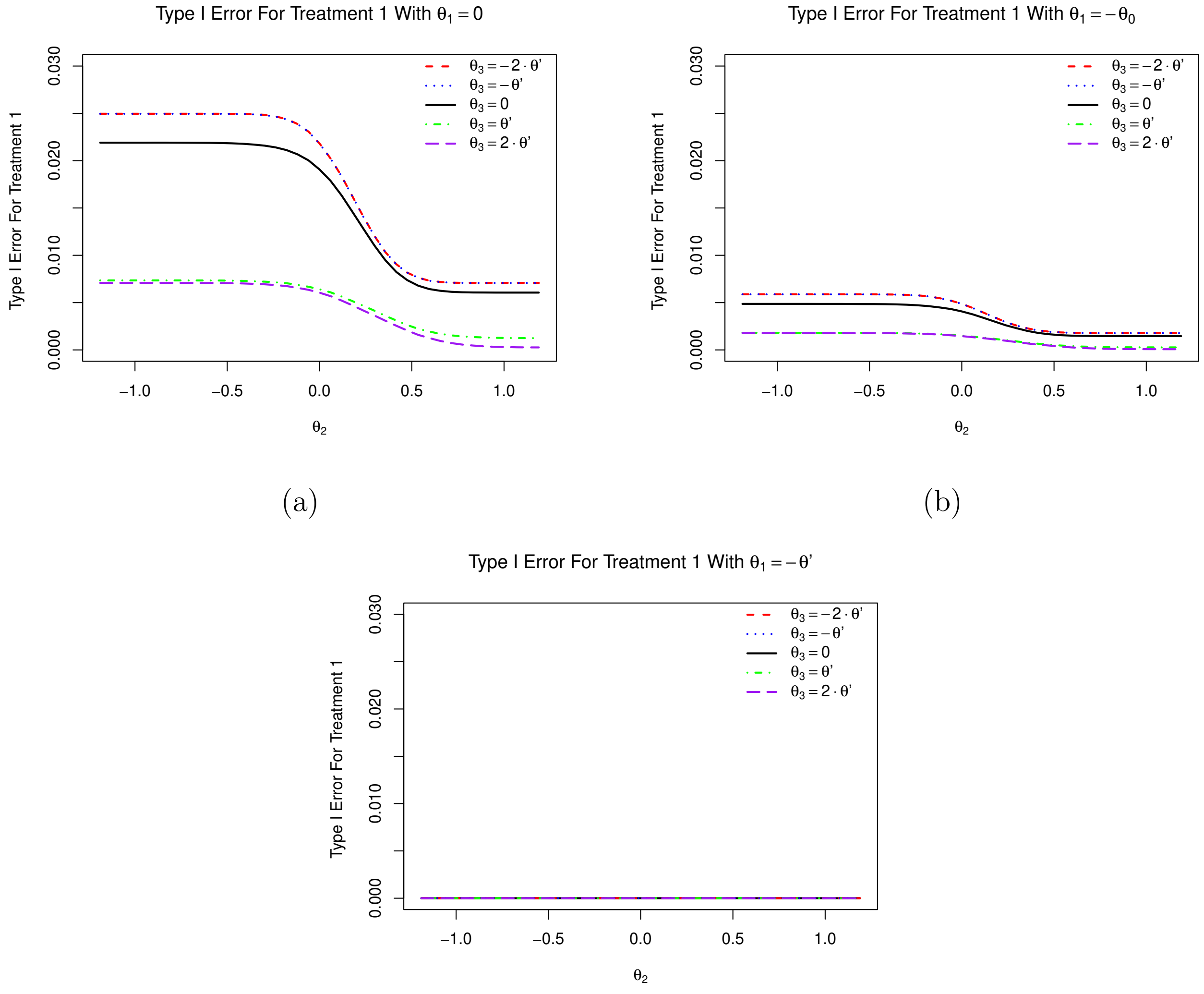

Figure 1: Type I error for treatment 1 under different values of θ_1, θ_2 and θ_3.
